# Cluster of Differentiation 10 Positive Stromal Sarcoma of Breast: A Diagnostic Challenge

**DOI:** 10.7759/cureus.5143

**Published:** 2019-07-15

**Authors:** Sharma Shruti, Pooja Gupta, Akanksha Malik, Amar Bhatnagar, Fouzia Siraj

**Affiliations:** 1 Pathology, National Institute of Pathology, New Delhi, IND; 2 Surgery, Vardhaman Mahavir Medical College and Safdarjung Hospital, New Delhi, IND

**Keywords:** breast sarcoma, mesenchymal tumor, immunohistochemistry, malignant phylloides tumor, stromal sarcoma, cd10

## Abstract

Stromal sarcomas of the breast are a group of rare and heterogeneous tumors which mimic malignant phylloides tumor and metaplastic carcinoma histologically. These tumors have been reported in the literature mostly in form of small retrospective case series and case reports, making it difficult to characterize their exact histopathological spectrum and management strategy. Our patient was a 65-year-old female who presented with a 3-month history of a lump in the left breast. Examination revealed an immobile mass in upper and outer quadrant of left breast, 5 x 4 cm in diameter. Trucut biopsy revealed sheets of atypical spindle cells. She underwent a modified radical mastectomy. On histopathology, we found malignant mesenchymal tumor positive for vimentin and cluster of differentiation 10 (CD10). Thus, a rare diagnosis of CD10 positive mammary stromal sarcoma was established. The case has been reported for its rarity and to highlight the importance of a meticulous histopathological examination for excluding close differentials.

## Introduction

Undifferentiated mammary sarcoma (UMS) is a rare malignant tumor arising from the mesenchymal tissue of the mammary gland [1]. Stromal sarcomas of the breast are a group of rare and heterogeneous tumors that represent less than one percent of all primary breast malignancies and less than five percent of all soft tissue sarcomas [2]. They are at an increased risk for local or distant recurrence, accounting for a relatively poor prognosis. Histologically, malignant phylloides tumor and metaplastic carcinoma are their close differentials. The exact histogenesis of these tumors is not clear, with some authors postulating their origin from the specialized stroma of the breast. These tumors have been described in the existing literature mostly in the form of small retrospective case series and case reports; hence, our knowledge about their histopathological spectrum continues to evolve.

## Case presentation

**Figure 1:**
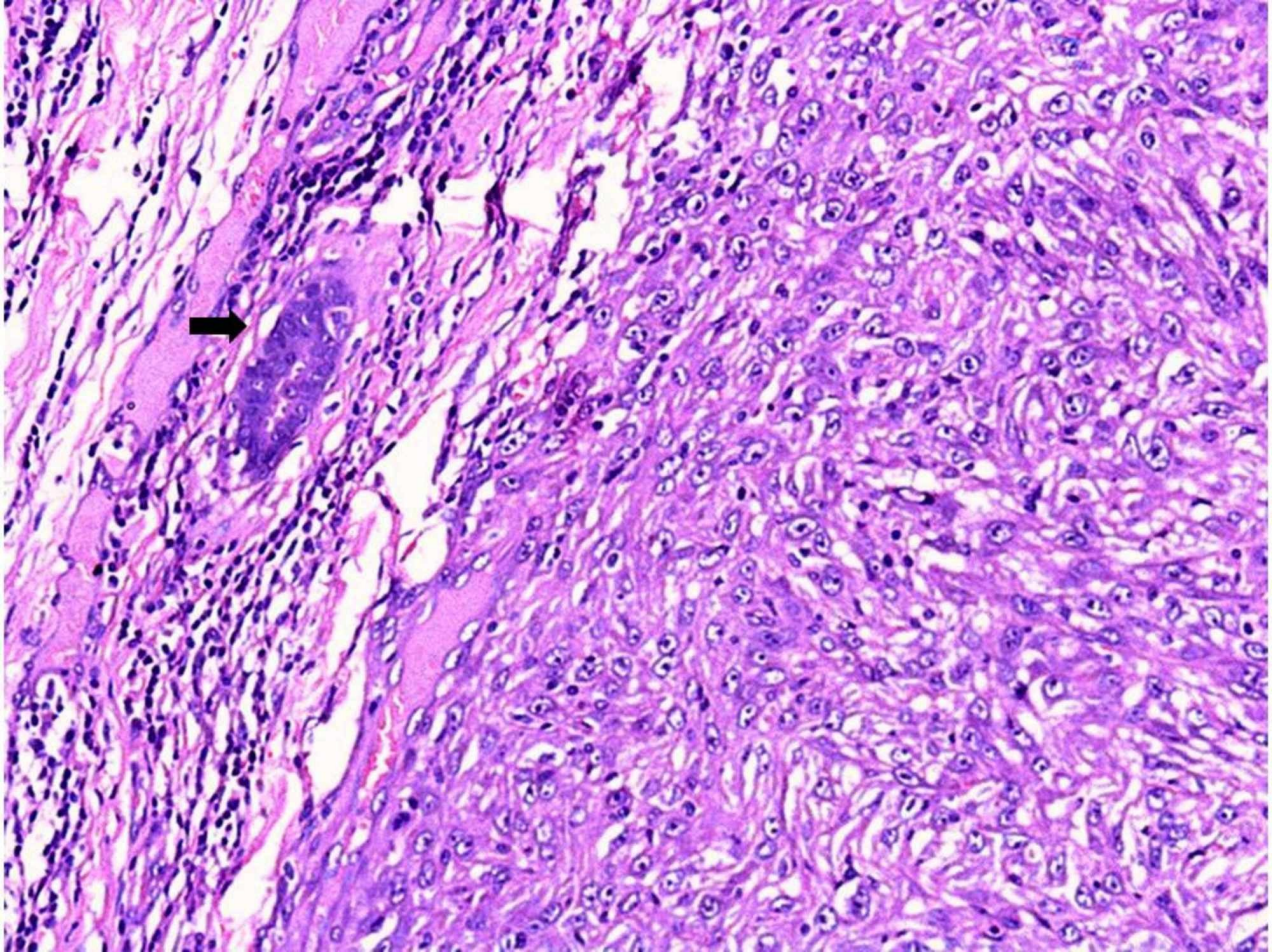
Photomicrograph of a well-circumscribed tumor composed of oval to spindle cells arranged in interlacing bundles, fascicles, and whorls (H& E x100) A benign duct is observed in the periphery [arrow].

A 65-year-old female presented with a lump in the left breast for the past three months. There was no history of nipple discharge, axillary lump or symptoms suggestive of distant metastases. She denied a past history of malignancy or irradiation and her family history was unremarkable. Examination revealed an immobile mass in upper and outer quadrant of left breast, 5 x 4 cm in diameter. There was no evidence of regional lymphadenopathy or palpable mass in the opposite breast. Ultrasonography revealed a heterogeneous lesion measuring 5.5 × 4.0 cm. A trucut biopsy was performed, the microscopic examination of which showed only atypical spindle cells. Based on the clinical and pathological profile, she underwent a modified radical mastectomy. On gross examination, a grey-white lesion measuring 5 × 4 × 4 cm with areas of necrosis and cystic degeneration was seen. Twelve lymph nodes measuring 0.5-1.5 cm were also isolated from the axillary tail. Histopathological examination revealed a well-circumscribed tumor composed of oval to spindle cells arranged in interlacing bundles, fascicles, and whorls. The absence of epithelial elements within the tumor was noteworthy; we could appreciate an occasional benign duct outside the tumor tissue (Figure 1).

**Figure 2:**
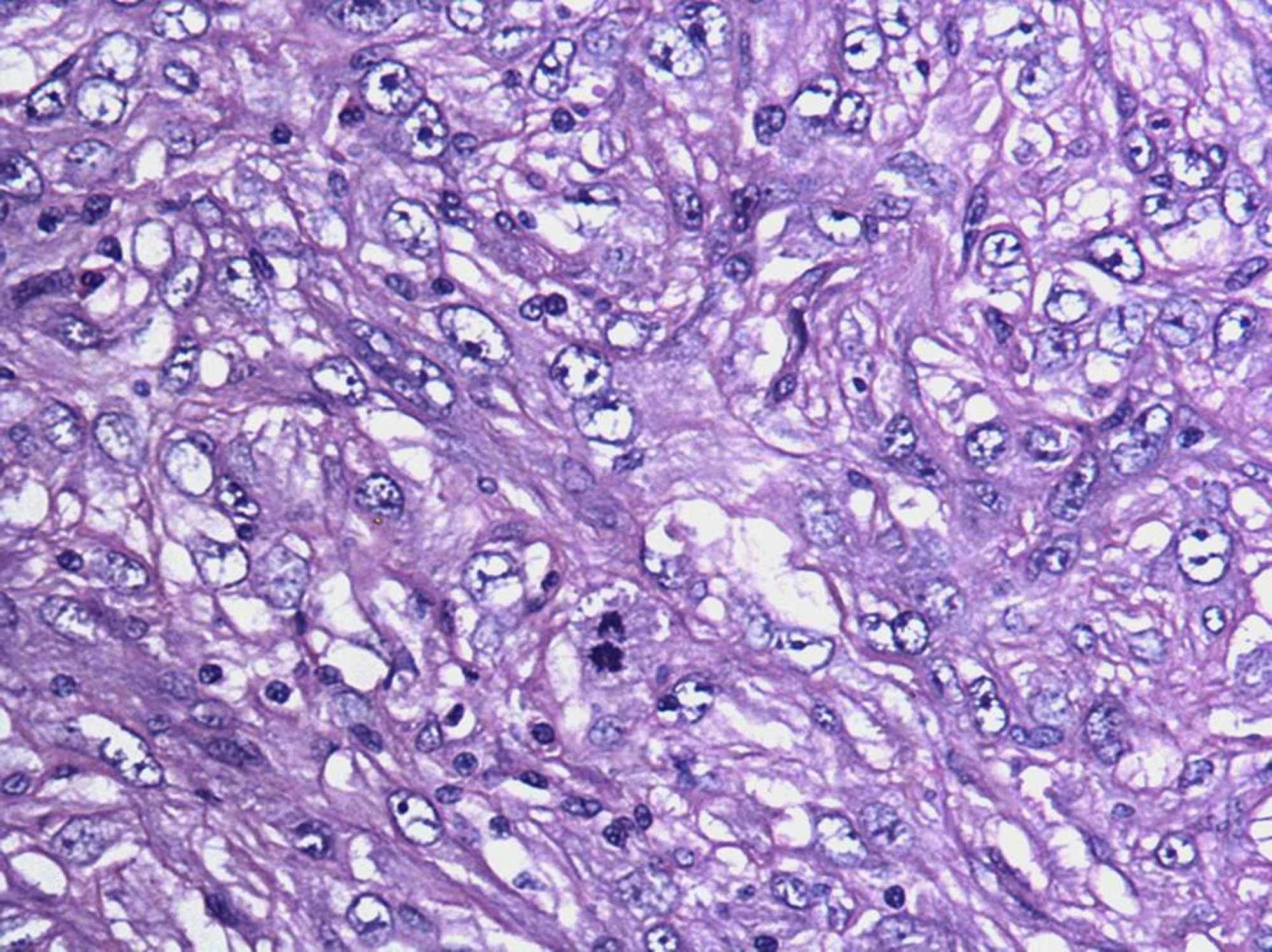
Photomicrograph showing nuclear atypia, brisk mitoses and lack of epithelial elements within the tumor (H&E x200)

No broad papillae or leaf-like structures were observed in the tumor tissue despite extensive sampling. The spindle cells showed marked atypia with nuclear pleomorphism and occasional cytoplasmic vacuolation. Areas of necrosis and brisk mitotic activity (7/10 high-power fields) were evident (Figure 2).

**Figure 3:**
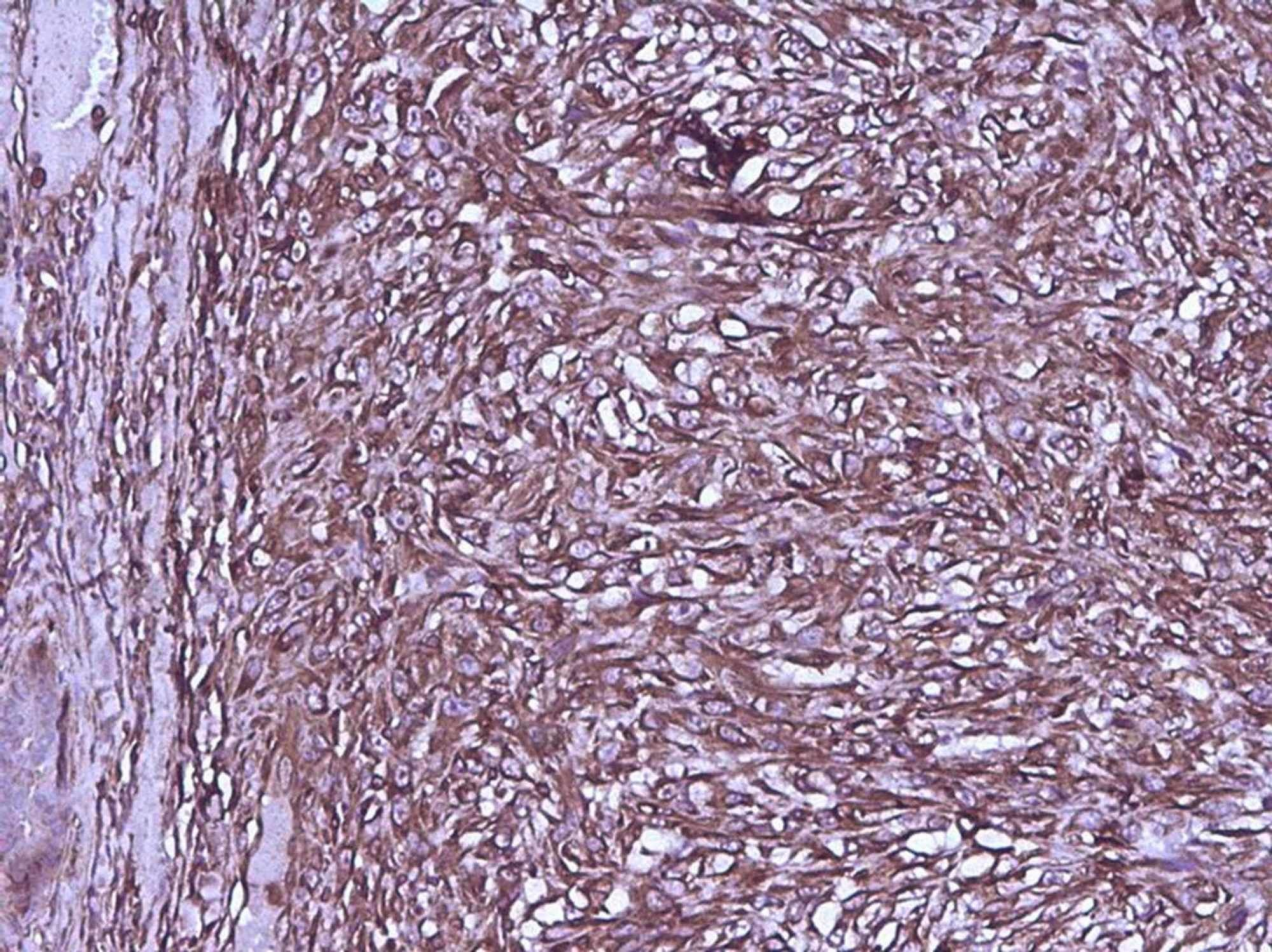
Photomicrograph showing immunohistochemical positivity of tumor cells for vimentin (x200)

**Figure 4:**
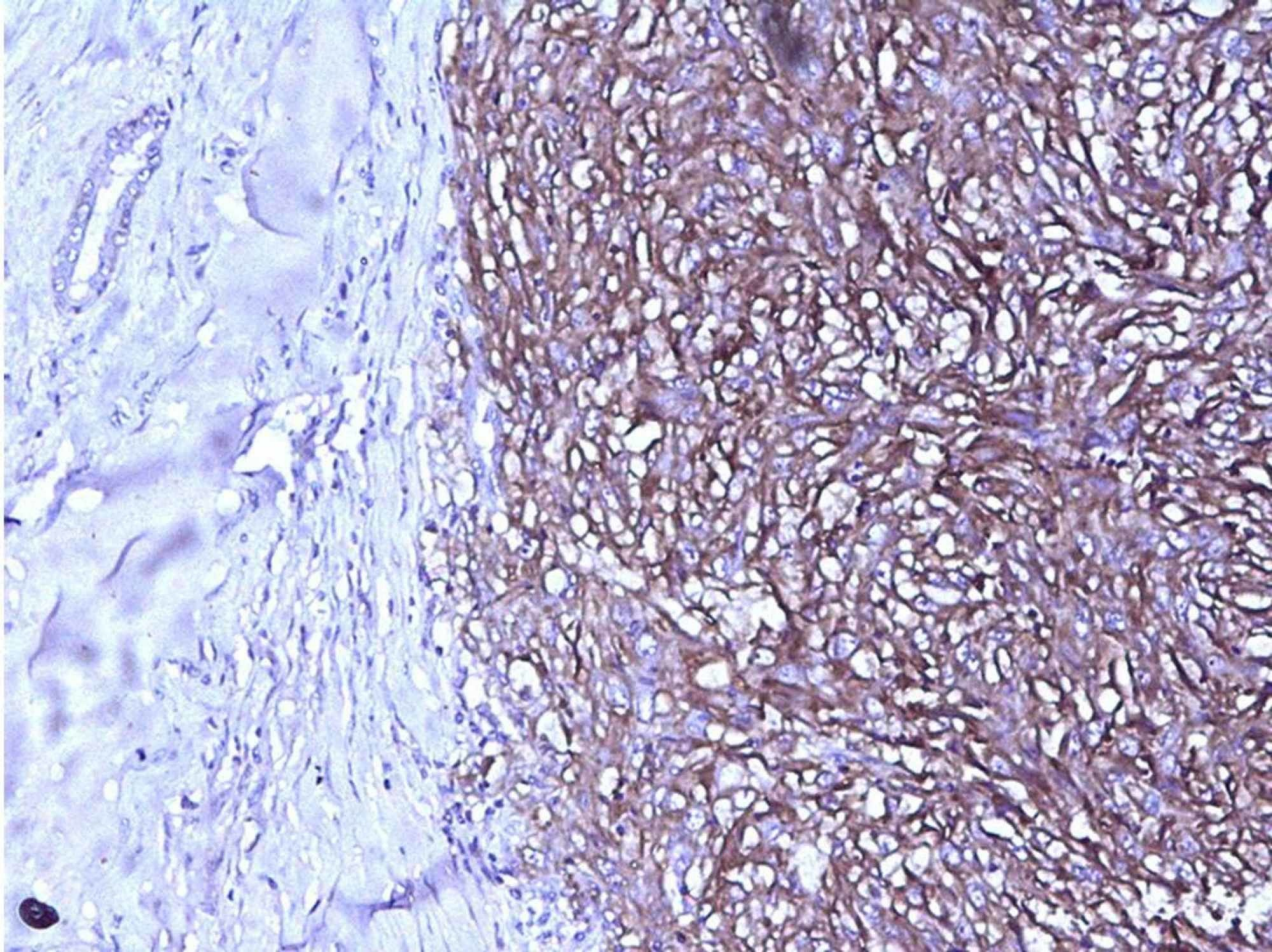
Photomicrograph showing immunohistochemical positivity of tumor cells for CD10 (x200)

All the peripheral and deep resected margins were free from the tumor. No lymphovascular or neural invasion was seen. All resected lymph nodes showed reactive follicular hyperplasia without evidence of any metastatic pathology. We considered following differentials: stromal sarcoma, phylloides tumor, metaplastic carcinoma, pleomorphic leiomyosarcoma, pleomorphic rhabdomyosarcoma, malignant peripheral nerve sheath tumor (MPNST) and undifferentiated pleomorphic sarcoma. A panel of immunohistochemical markers comprising of vimentin, cytokeratin (CK), cluster of differentiation 10 (CD10), CD34, estrogen receptor (ER), progesterone receptor (PR), human epidermal growth factor receptor 2 (Her-2 neu), smooth muscle actin (SMA), desmin, myogenin, S-100 and CD68 was performed. Tumor cells were positive for vimentin and CD10 and negative for all other markers (Figures 3, 4).

Based on histomorphology and immunohistochemistry, a diagnosis of CD10 positive stromal sarcoma of the breast was considered. She had good postoperative recovery and was asymptomatic till the last follow-up visit three months after the surgery. Since the role of adjuvant chemo and radiotherapy for these tumors is not clear, the patient opted against their use.

## Discussion

Stromal sarcoma of breast is a rare pathology which accounts for less than one percent of all breast malignancies. The age of presentation varies; however, it is most commonly reported in women in their fifth or sixth decade of life [3]. Clinically, such tumors present as a rapidly growing breast lump, which may reach large proportions. They tend to spread by local invasion and hematogenous route and carry an overall poor prognosis. The predisposing conditions for their development are not exactly clear. These tumors may develop de novo or secondary to a) external beam radiation of the breast or chest wall, b) chronic lymphedema of the breast and arm after treatment for another malignancy (especially angiosarcoma), c) pre-existing fibroadenomata, and d) hereditary diseases, like neurofibromatosis or Li-Fraumeni syndrome [4]. There was no history of any such predisposing condition in our case.

Histologically, mammary stromal sarcomas are high-grade undifferentiated tumors that do not fit into a specific category of any soft tissue sarcomas. Microscopically, they are characterized by the presence of spindle cells with pleomorphic nuclei, brisk mitotic activity and immunohistochemical positivity for vimentin and CD10, and negativity for CD34 [5,6]. These tumors mimic other high-grade soft tissue sarcomas to an extent that a correct diagnosis without the aid of immunohistochemistry is nearly impossible. The various differentials of mammary stromal sarcomas have been discussed in Table 1.

**Table 1:** Differential diagnosis of breast tumors with sarcomatous morphology CD: Cluster of differentiation; SMA: Smooth muscle actin; CK: Cytokeratin; MyoD1: Myogenic differentiation 1; MPNST: Malignant peripheral nerve sheath tumor

Differential diagnosis	Histopathological features	Immunohistochemical features
Stromal sarcoma	Oval to spindle cells, nuclear atypia and brisk mitoses with lack of epithelial elements	Vimentin+, CD10+, CD34-, SMA-
Malignant phylloides tumor	Stromal hypercellularity, cellular atypia, high mitotic rate along with the presence of normal ductal elements	Vimentin+, CD34+/-, SMA+/-
Metaplastic carcinoma	Poorly differentiated ductal carcinoma with high-grade sarcomatous component	CK(Epithelial)+, Vimentin(Mesenchymal)+
Pleomorphic leiomyosarcoma	Fascicles of atypical spindle cells with cigar-shaped hyperchromatic nuclei and eosinophilic cytoplasm	SMA and Desmin+, CD10-
Pleomorphic rhabdomyosarcoma	Large, pleomorphic rhabdomyoblasts with cytoplasmic cross-striations	Desmin+, Myogenin+, MyoD1+, CD10-
MPNST	Cellular tumor characterized by pleomorphic cells with wavy nuclei, prominent mitotic activity and areas of tumor necrosis	S-100+
Undifferentiated pleomorphic sarcoma	Malignant proliferation of giant cells, histiocytes, fibroblasts, and myofibroblasts arranged in a storiform pattern.	Vimentin+, CD68+, CD10-

Phylloides tumor of breast is easily recognized by its characteristic leaf-like biphasic pattern; however, cases with stromal overgrowth may lack these leaf-like structures. Immunohistochemical staining for CD34 is important in such cases as these tumors are frequently positive for this marker [7]. Mammary stromal sarcomas also need to be differentiated from sarcomatoid carcinoma. These tumors are generally positive for basal cell CK (CK5, CK14), and p63. Leiomyosarcoma also shares similar histopathological features with mammary stromal sarcoma. However, in contrast to mammary stromal sarcoma, leiomyosarcoma is immunonegative for CD10 and positive for desmin [7].

CD10 is a marker for stromal cells and is important in prognosis and treatment of invasive breast carcinoma [8, 9]. It is also consistently expressed in myoepithelial cells during development and after maturation of breast tissue. The immunophenotype of CD10 expressing sarcomas suggests that these neoplasms represent a variant of mammary sarcoma with myoepithelial features. CD10 expression is associated with a high grade and estrogen receptor negativity [10]. Further, stromal CD10 expression in invasive breast carcinoma is found to be associated with decreased patient survival; hence, it constitutes a clinically important prognostic marker and a potential target for the development of novel therapies. Surgery remains the best therapeutic option for stromal sarcoma of breast. The use of adjuvant radiotherapy and chemotherapy has been met with conflicting results in various studies; hence, their role in management remains unclear [11]. The risk of disease recurrence is high, accounting for a relatively poor prognosis. The five-year disease free survival and overall survival have been reported at 44-66% and 49-67% in various studies [11].

## Conclusions

CD10 positive mammary stromal sarcomas are an extremely rare group of tumors which closely mimic other pathologies like malignant phylloides tumors and metaplastic carcinoma of breast. We have reported this case for its rarity and to highlight the importance of a meticulous histopathological and immunohistochemical examination in excluding the close differentials. There is a need for future functional studies elucidating the signaling mechanisms causing CD10 overexpression in mammary stromal sarcomas.
